# Secondary bonding in di­methyl­bis­(morpholine-4-carbodi­thio­ato-κ^2^ *S*,*S*′)tin(IV): crystal structure and Hirshfeld surface analysis

**DOI:** 10.1107/S2056989017006855

**Published:** 2017-05-12

**Authors:** Nordiyana Binti Zaldi, Rusnah Syahila Duali Hussen, See Mun Lee, Nathan R. Halcovitch, Mukesh M. Jotani, Edward R. T. Tiekink

**Affiliations:** a Department of Chemistry, University of Malaya, 50603 Kuala Lumpur, Malaysia; b Research Centre for Crystalline Materials, School of Science and Technology, Sunway University, 47500 Bandar Sunway, Selangor Darul Ehsan, Malaysia; c Department of Chemistry, Lancaster University, Lancaster LA1 4YB, United Kingdom; d Department of Physics, Bhavan’s Sheth R. A. College of Science, Ahmedabad, Gujarat 380 001, India; e Research Centre for Chemical Crystallography, School of Science and Technology, Sunway University, 47500 Bandar Sunway, Selangor Darul Ehsan, Malaysia

**Keywords:** crystal structure, organotin, di­thio­carbamate, tetrel bonding, Hirshfeld surface analysis

## Abstract

The title compound, [Sn(CH_3_)_2_(C_5_H_8_NOS_2_)_2_], has the Sn^IV^ atom bound by two methyl groups which lie over the weaker Sn—S bonds formed by two asymmetrically chelating di­thio­carbamate ligands so that the coordination geometry is skew-trapezoidal bipyramidal. The most prominent feature of the mol­ecular packing are secondary Sn⋯S inter­actions [Sn⋯S = 3.5654 (7) Å] that lead to centrosymmetric dimers. These are connected into a three-dimensional architecture *via* methyl­ene-C—H⋯S and methyl-C—H⋯O(morpholino) inter­actions. The Sn⋯S inter­actions are clearly evident in the Hirshfeld surface analysis of the title compound along with a number of other inter­molecular contacts.

## Abstract 2

In (CH_3_)_2_Sn[S_2_CN(CH_2_CH_2_)_2_O]_2_, a skew-trapezoidal bipyramidal coordination geometry based on a C_2_S_4_ donor set is found. Secondary Sn⋯S inter­actions lead to centrosymmetric dimeric aggregates in the crystal.

## Chemical context   

Both binary tin and organotin di­thio­carbamates, *R_n_*Sn(S_2_CN*RR*′)_*m*_ for *n* + *m* = 4, are well known to exhibit potential biological properties, *e.g*. anti-cancer (Ferreira *et al.*, 2014 ▸), anti-fungal (Yu *et al.*, 2014 ▸) and anti-microbial (Ferreira *et al.*, 2012 ▸), as well to serve as useful mol­ecular precursors for the generation of ‘SnS’ nanomaterials (Kevin *et al.*, 2015 ▸). The structural chemistry of this class of compound has also attracted considerable inter­est over the years owing to the occurrence of significant structural diversity observed in seemingly closely related compounds (Tiekink, 2008 ▸). As a case in point and related to the title compound, [Sn(CH_3_)_2_(C_5_H_8_NOS_2_)_2_] (I), reported herein, are the variations in mol­ecular structure observed for the diorganotin bis­(di­thio­carbamate)s as discussed in the recent literature (Muthalib *et al.*, 2014 ▸; Mohamad *et al.*, 2016 ▸, 2017 ▸). These *R*_2_Sn(S_2_CN*RR*’)_2_ structures are known to adopt four distinct coordination geometries with the majority being skew-trapezoidal bipyramidal or octa­hedral, each based on C_2_S_4_ donor sets. Fewer examples are known for five-coordinate, trigonal–bipyramidal species, *e.g*. (*t*-Bu)_2_Sn(S_2_CNMe_2_)_2_ in which one di­thio­carbamate ligand is monodentate (Kim *et al.*, 1987 ▸), and seven-coordinate, penta­gonal–bipyramidal, *e.g*. [MeOC(=O)CH_2_CH_2_]_2_Sn(S_2_CNMe)_2_ where the carbonyl-O atom of one Sn-bound organic substituent is also coordinating the tin atom (Ng *et al.*, 1989 ▸). This last example is of inter­est as it demonstrates tin may in fact increase its coordination number by additional inter­actions. When additional inter­actions of this type occur inter­molecularly, they are termed secondary bonding or tetrel bonding as a Group IV element, tin, is involved (Alcock, 1972 ▸; Marín-Luna *et al.*, 2016 ▸; Tiekink, 2017 ▸). Generally, secondary inter­actions do not occur for *R*_2_Sn(S_2_CN*RR*’)_2_ structures as the strong chelating ability of the di­thio­carbamate ligand reduces the Lewis acidity of the tin atom. However, in (I) such secondary Sn⋯S inter­actions do in fact occur. In a continuation of work in this area, herein the synthesis and crystal and mol­ecular structures of (I) are described as well as an analysis of the Hirshfeld surface with a particular emphasis on investigating the role of the secondary Sn⋯S inter­action.
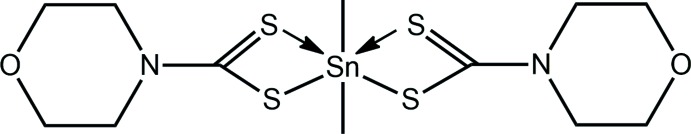


## Structural commentary   

**Figure 1:**
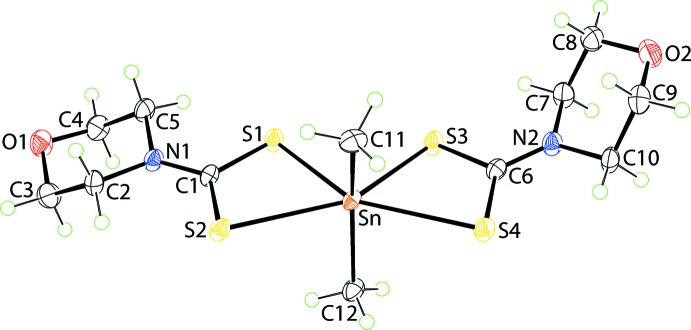
The mol­ecular structure of (I), showing the atom-labelling scheme and displacement ellipsoids at the 50% probability level.

The Sn^IV^ atom in the title compound (I), Fig. 1 ▸, adopts one of the common coordination geometries found for *R*_2_Sn(S_2_CN*RR*’)_2_ mol­ecules, *i.e*. skew-trapezoidal bipyramidal rather than octa­hedral (Tiekink, 2008 ▸). This arises as the chelating di­thio­carbamate ligands have asymmetric Sn—S bond lengths, Table 1 ▸. The values of Δ(Sn—S) = [*d*(Sn—S_long_) − *d*(Sn—S_short_] for the S1- and S3-di­thio­carbamate ligands are approximately the same at 0.35 Å, but the comparable bonds formed by the S3-di­thio­carbamate ligand are systematically longer than those formed by the S1-di­thio­carbamate ligand by approximately 0.02 Å, Table 1 ▸. The asymmetry in the Sn—S bond lengths is reflected in the disparity in the associated C—S bond lengths with the sulfur atom forming the longer Sn—S bond being involved in the significantly shorter, by approx­imately 0.05 Å, C—S bond, Table 1 ▸. Consistent with the skew-trapezoidal bipyramidal geometry about the Sn^IV^ atom, the Sn-bound methyl substituents are directed over the longer Sn—S bonds and define an angle of 148.24 (11)° at the tin atom. The angle subtended at the tin atom by the strongly bound sulfur atoms of 85.878 (19)° is significantly less than that formed by the weakly bound sulfur atoms, *i.e*. 143.066 (18)°, and is largely responsible for the formation of the skew-trapezoidal plane about the tin atom.

## Supra­molecular features   

**Figure 2:**
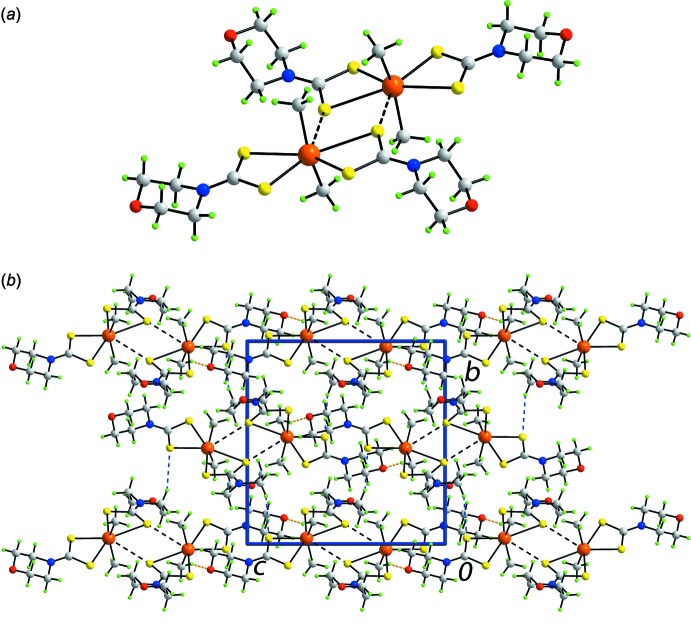
The mol­ecular packing in (I), showing (*a*) a supra­molecular dimer sustained by Sn⋯S secondary inter­actions shown as black dashed lines and (*b*) a view of the unit-cell contents in projection down the *a* axis. The C—H⋯S and C—H⋯O inter­actions are shown as orange and blue dashed lines, respectively.

An inter­esting feature of the mol­ecular packing in (I) is the formation of a supra­molecular dimer sustained by Sn⋯S secondary inter­actions, as shown in Fig. 2 ▸ *a*, where two long edges of the translationally displaced trapezoidal planes approach each other to form the inter­actions. Here, Sn⋯S4^i^ is 3.5654 (7) Å, which is approximately 0.4 Å shorter than the sum of the van der Waals radii of Sn and S of 3.97 Å (Bondi, 1964 ▸); symmetry operation (i): 1 − *x*, 1 − *y*, 1 − *z*. Connections between the dimeric aggregates are of the type methyl­ene-C—H⋯S and methyl-C—H⋯O(morpholino), Table 2 ▸, and these inter­actions combine to generate a three-dimensional architecture, Fig. 2 ▸ *b*.

## Hirshfeld surface analysis   

**Figure 3:**
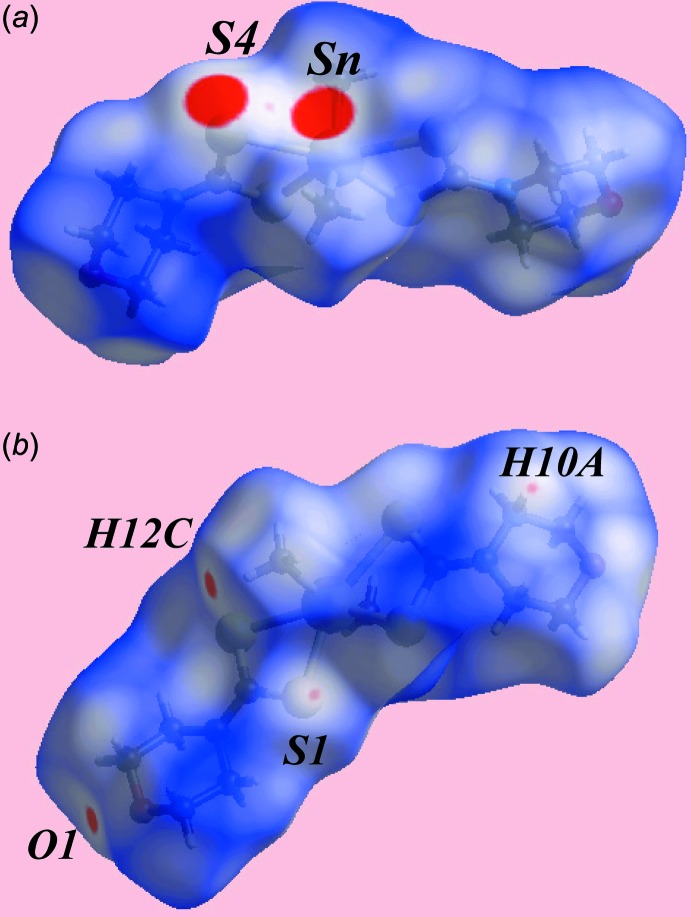
Two views of the Hirshfeld surface for (I) plotted over *d*_norm_ in the range −0.050 to 1.780 au.

**Figure 4:**
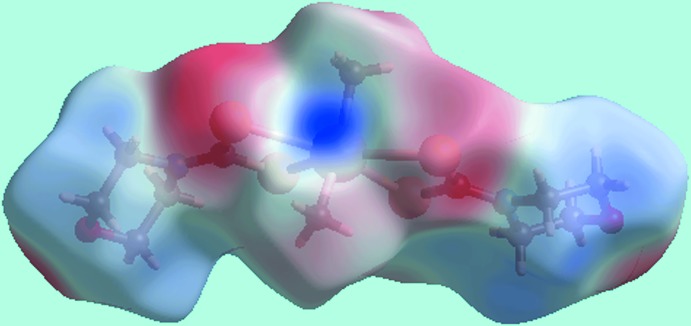
A view of Hirshfeld surface for (I) mapped over the calculated electrostatic potential in the range −0.053 to +0.078 au. The red and blue regions represent negative and positive electrostatic potentials, respectively.

**Figure 5:**
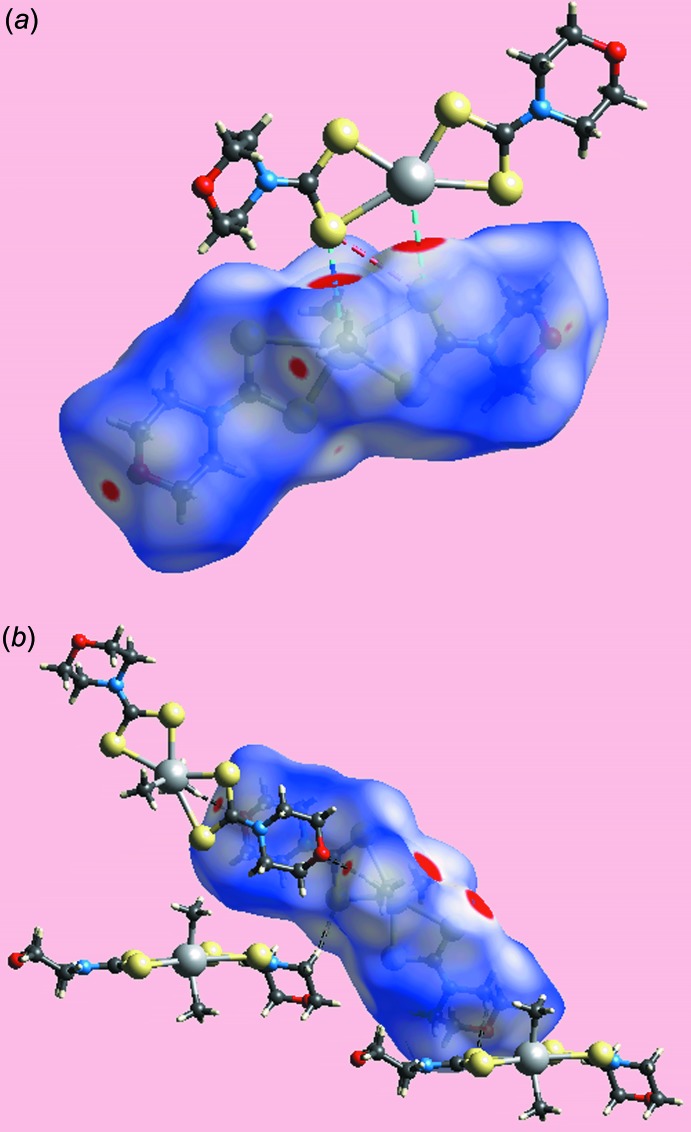
Views of Hirshfeld surfaces mapped over *d*_norm_ about a reference mol­ecule showing (*a*) secondary Sn⋯S/S⋯Sn and S⋯S contacts by sky-blue and red dashed lines, respectively and (*b*) C—H⋯O and C—H⋯S inter­actions by black dashed lines

The Hirshfeld surfaces calculated on the structure of (I) also provide insight into the supra­molecular association through secondary Sn⋯S, S⋯S and other contacts, and was performed as per recent publications on related organotin di­thio­carbamate structures (Mohamad *et al.*, 2017 ▸, 2016 ▸). The broad, bright-red spots appearing near the Sn and S4 atoms on the Hirshfeld surfaces mapped over *d*_norm_ in Fig. 3 ▸ *a* indicate the formation of the supra­molecular dimer through secondary Sn⋯S contacts. On the Hirshfeld surface mapped over electrostatic potential in Fig. 4 ▸, these inter­actions are represented by the blue and red regions around these atoms, respectively. The faint-red spot appearing between the above bright-red spots near the S4 atom indicates the short inter-atomic S⋯S contact, Table 3 ▸, between S4 atoms lying on diagonally opposite vertices of a parallelogram formed by symmetry-related Sn and S4 atoms, Fig. 5 ▸ *a*. The pair of bright-red spots appearing near the methyl-H12*C* and morpholine-O1 atoms in Fig. 3 ▸ *b* represent the respective donor and acceptor atoms of the C12—H⋯O1 inter­action. The comparatively weaker methyl­ene-C10—H⋯S1 inter­action is viewed as a pair of faint-red spots near these atoms in Fig. 3 ▸ *b*. It is important to note from the immediate environments about a reference mol­ecule within *d*_norm_-mapped Hirshfeld surfaces highlighting inter­molecular inter­actions in Fig. 5 ▸ that the secondary Sn⋯S and S⋯S contacts are on one side of the Hirshfeld surface while the atoms participating in C—H⋯O and C—H⋯S inter­actions are on the other side of the surface.

**Figure 6:**
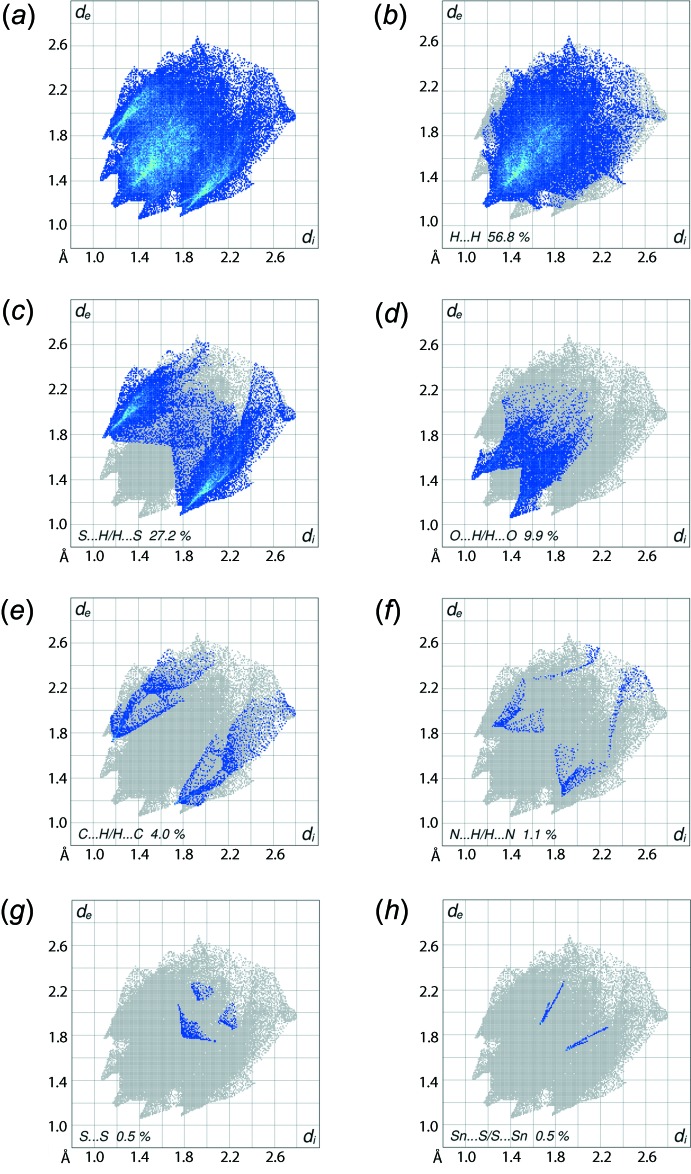
(*a*) The full two-dimensional fingerprint plot for (I) and fingerprint plots delineated into (*b*) H⋯H, (*c*) S⋯H/H⋯S, (*d*) O⋯H/H⋯O, (*e*) C⋯H/H⋯C, (*f*) N⋯H/H⋯H, (*g*) Sn⋯S/S⋯Sn and (*h*) S⋯S contacts.

The overall two-dimensional fingerprint plot, Fig. 6 ▸ *a*, and those delineated into H⋯H, S⋯H/H⋯S, O⋯H/H⋯O, C⋯H/H⋯C, N⋯H/H⋯N, Sn⋯S/S⋯Sn and S⋯S contacts (McKinnon *et al.*, 2007 ▸) are illustrated in Fig. 6 ▸ *b*–*h*, respectively; the relative contributions from the various contacts to the Hirshfeld surfaces are summarized in Table 4 ▸.

In the fingerprint plot delineated into H⋯H contacts, Fig. 6 ▸ *b*, the points forming the single short peak at *d*_e_ + *d*_i_ < 2.4 Å are indicative of the short inter-atomic H⋯H contact listed in Table 3 ▸. The involvement of S1 in the C—H⋯S inter­action and other sulfur atoms in short inter-atomic S⋯H/H⋯S contacts, Table 3 ▸, results in an overall 27.2% contribution to the Hirshfeld surface. In the fingerprint plot delineated into S⋯H/H⋯S contacts, Fig. 6 ▸ *c*, they appear as overlapping donor–acceptor regions showing corners and a pair of greenish regions of greater intensity having short spikes at *d*_e_ + *d*_i_ ∼ 2.9 Å. The C—H⋯O contact is evident from the two-dimensional fingerprint plot delineated into O⋯H/H⋯O contacts, Fig. 6 ▸ *d*, as the pair of tips at *d*_e_ + *d*_i_ ∼ 2.5 Å in the forceps-like distribution. The short inter-atomic O⋯H/H⋯O contacts, Table 3 ▸, in the plot appear as faint-green points in a slightly scattered form emanating from *d*_e_ + *d*_i_ ∼ 2.9 Å. The pair of short spikes at *d*_e_ + *d*_i_ < 2.9 Å overlapping on the well separated donor and acceptor regions in the fingerprint plot delineated into C⋯H/H⋯C contacts, Fig. 6 ▸ *e*, indicate the influence of short inter-atomic C⋯H/H⋯C contacts, Table 3 ▸. The presence of secondary Sn⋯S and short S⋯S contacts in the structure is also confirmed from the respective plots through the distribution of points as a pair of thin line segments, Fig. 6 ▸ *f*, and a triangle, Fig. 6 ▸ *g*, respectively, having minimum *d*_e_ + *d*_i_ distances at around 3.5 Å and 3.6 Å, respectively. The 1.1% contribution from N⋯H/H⋯N contacts, Fig. 6 ▸ *h*, to the Hirshfeld surface reflects an insignificant influence upon the mol­ecular packing as the inter-atomic separations are greater than the sum of the respective van der Waals radii.

## Database survey   

**Figure 7:**
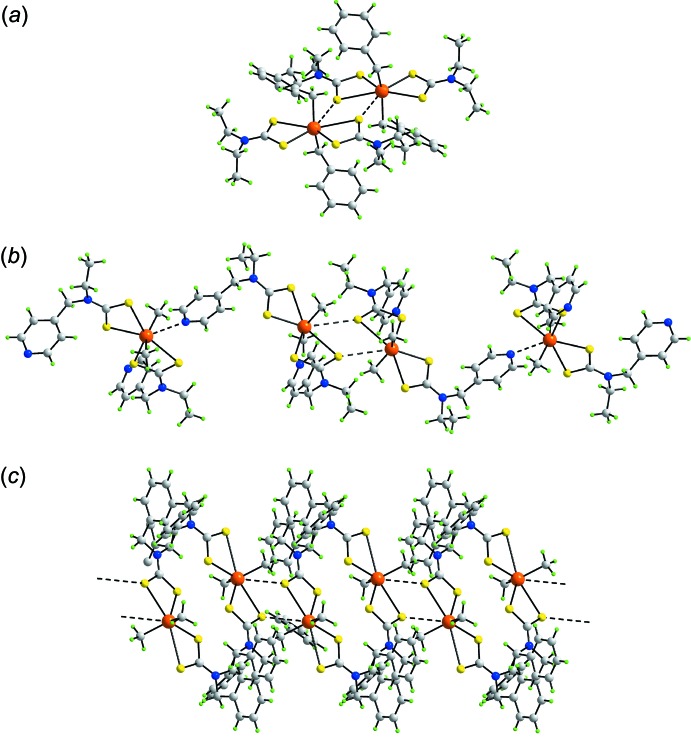
Supra­molecular aggregation sustained by secondary Sn⋯S inter­actions (black dashed lines) leading to (*a*) dimeric aggregates in (PhCH_2_)_2_Sn(S_2_CNEt_2_)_2_, (*b*) four-mol­ecule aggregates in Me_2_Sn(S_2_CN(Et)CH_2_C_6_H_4_N-4)_2_ and (*c*) linear supra­molecular chain in {Me_2_SnS_2_CN(CH_2_Ph)CH_2_(1,3-C_6_H_3_)CH_2_(PhCH_2_)NCS_2_SnMe_2_}_2_.

The Cambridge Crystallographic Database (Groom *et al.*, 2016 ▸) contains over 110 mol­ecules of the general formula *R*_2_Sn(S_2_CN*RR*’)_2_. Of these, 12 feature secondary Sn⋯S inter­actions which, with (I), means approximately 10% of all *R*_2_Sn(S_2_CN*RR*’)_2_ structures have Sn⋯S secondary inter­actions. Selected geometric details for the 13 structures are collated in Table 5 ▸. The Sn⋯S inter­actions assemble mol­ecules in their crystals into three distinct structural motifs. The common motif, *A*, is a dimeric aggregate disposed about a centre of inversion, as is in (I), and is found in the majority of crystals, *i.e*. nine. This motif is illustrated in Fig. 7 ▸ *a* for (PhCH_2_)_2_Sn(S_2_CNEt_2_)_2_ (Yin *et al.*, 2003 ▸). A second zero-dimensional motif, *B*, is also known and is readily related to *A*. In the structure of Me_2_Sn(S_2_CN(Et)CH_2_C_6_H_4_N-4)_2_ (Barba *et al.*, 2012 ▸), two independent mol­ecules comprise the asymmetric unit. One of these self-assembles about a centre of inversion as for motif *A*. The nitro­gen atom of each pendent 4-pyridyl group of the dimeric aggregate thus assembled inter­acts with the tin atom of the second independent mol­ecule *via* a Sn⋯N inter­action to form the four-mol­ecule aggregate shown in Fig. 7 ▸ *b*. The final three mol­ecules are binuclear owing to the presence of bis­(di­thio­carbamate) ligands and self-assemble into supra­molecular chains. In {Me_2_SnS_2_CN(CH_2_Ph)CH_2_(1,3-C_6_H_3_)CH_2_(PhCH_2_)NCS_2_SnMe_2_}_2_ (Santacruz-Juárez *et al.*, 2008 ▸), the mol­ecule is situated about a centre of inversion and each tin atom forms an Sn⋯S contact to generate a linear, supra­molecular chain, motif *C*, Fig. 7 ▸ *c*. A variation is seen in the crystal of Me_2_SnS_2_CN(CH_2_CH_2_-*i*-Pr)CH_2_(1,3-C_6_H_3_)CH_2_(PhCH_2_)NCS_2_SnMe_2_}_2_, where there are two independent, centrosymmetric mol­ecules in the asymmetric unit. Here, the resulting supra­molecular chain is twisted (Santacruz-Juárez *et al.*, 2008 ▸) and is assigned as motif *C*′.

The common feature of all motifs listed in Table 5 ▸ is that it is one of the weakly bound sulfur atoms that forms the secondary Sn⋯S inter­action. Further, the tin-bound groups are relatively sterically unencumbered, allowing for the close approach of sulfur donors to the tin atoms. There are no geometric correlations. However, reflecting the weak nature of these inter­actions, the sulfur atom forming the Sn⋯S contact does not necessarily form the weaker of the Sn—S_long_ inter­actions in each mol­ecule. The range of Sn⋯S distances spans nearly 0.5 Å but, again, no correlations between these distances and the S_long_—Sn—S_long_ angles is apparent, *i.e*. it might be expected that the shorter Sn⋯S inter­actions would result in wider S_long_—Sn—S_long_ angles.

## Synthesis and crystallization   

All chemicals and solvents were used as purchased without purification, and all reactions were carried out under ambient conditions. The melting point was determined using an Electrothermal digital melting point apparatus and was uncorrected. The IR spectrum for (I) was obtained on a Perkin Elmer Spectrum 400 FT Mid-IR/Far-IR spectrophotometer in the range 4000 to 400 cm^−1^. The ^1^H NMR spectrum was recorded at room temperature in CDCl_3_ solution on a Jeol ECA 400 MHz FT–NMR spectrometer.

Sodium morpholine­dithio­carbamate (prepared from the reaction between carbon di­sulfide and morpholine (Merck) in the presence of sodium hydroxide; 1.0 mmol, 0.185 g) in methanol (20 ml) was added to di­methyl­tin dichloride (Merck, 1.0 mmol, 0.219 g) in methanol (10 ml). The resulting mixture was stirred and refluxed for 2 h. The filtrate was evaporated until an off-white precipitate was obtained. The precipitate was recrystallized from methanol solution by slow evaporation to yield colourless prisms. Yield: 0.305 g, 64.4%; m.p.: 448 K. IR (cm^−1^): 1465(*s*), 1423(*s*) ν(C—N), 1222(*s*) ν(C—O), 1110(*m*), 994(*s*) ν(C—S), 541(*m*) ν(Sn—C) cm^−1^. ^1^H NMR (CDCl_3_): 4.18 (*s*, 8H, CH_2_O), 3.77 (*s*, 8H, NCH_2_), 1.54 (*s*, 6H, -CH_3_).

## Refinement   

Crystal data, data collection and structure refinement details are summarized in Table 6 ▸. Carbon-bound H atoms were placed in calculated positions (C—H = 0.98–0.99 Å) and were included in the refinement in the riding-model approximation, with *U*_iso_(H) set to 1.2–1.5*U*_eq_(C). Owing to poor agreement, one reflection, *i.e*. (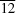 1 5), was omitted from the final cycles of refinement.

## Supplementary Material

Crystal structure: contains datablock(s) I, global. DOI: 10.1107/S2056989017006855/hb7675sup1.cif

Structure factors: contains datablock(s) I. DOI: 10.1107/S2056989017006855/hb7675Isup2.hkl

CCDC reference: 1548414

Additional supporting information:  crystallographic information; 3D view; checkCIF report

## Figures and Tables

**Table 1:** Selected geometric parameters (Å, °)

Sn—S1	2.5429 (6)	Sn—C12	2.111 (3)
Sn—S2	2.8923 (6)	C1—S1	1.747 (3)
Sn—S3	2.5649 (7)	C1—S2	1.702 (3)
Sn—S4	2.9137 (6)	C6—S3	1.750 (3)
Sn—C11	2.132 (3)	C6—S4	1.697 (3)
			
S1—Sn—S2	65.935 (19)	S2—Sn—C12	84.93 (8)
S1—Sn—S3	85.878 (19)	S3—Sn—S4	65.137 (18)
S1—Sn—S4	150.95 (2)	S3—Sn—C12	102.37 (8)
S1—Sn—C11	99.49 (8)	S3—Sn—C11	99.28 (8)
S1—Sn—C12	104.96 (8)	S4—Sn—C11	84.20 (8)
S2—Sn—S3	151.798 (18)	S4—Sn—C12	84.15 (7)
S2—Sn—S4	143.066 (18)	C11—Sn—C12	148.24 (11)
S2—Sn—C11	86.87 (8)		

**Table 2:** Hydrogen-bond geometry (Å, °)

*D*—H⋯*A*	*D*—H	H⋯*A*	*D*⋯*A*	*D*—H⋯*A*
C10—H10*A*⋯S1^i^	0.99	2.86	3.809 (3)	161
C12—H12*C*⋯O1^ii^	0.98	2.47	3.399 (4)	158

Symmetry codes: (i) 
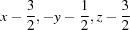
; (ii) 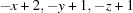.

**Table 3:** Summary of short inter-atomic contacts (Å) in (I)

Contact	distance	symmetry operation
S4⋯S4	3.5835 (10)	1 − *x*, 1 − *y*, −*z*
S2⋯H12*B*	2.99	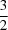 − *x*, 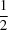 + *y*, 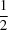 − *z*
S3⋯H5*B*	2.94	1 − *x*, 1 − *y*, 1 − *z*
S4⋯H11*C*	2.94	1 − *x*, 1 − *y*, − *z*
O2⋯H2*A*	2.63	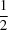 − *x*, − 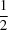 + *y*, 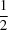 − *z*
O2⋯H5*B*	2.70	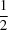 − *x*, − 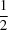 + *y*, 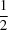 − *z*
C1⋯H3*A*	2.88	2 − *x*, 1 − *y*, 1 − *z*
C3⋯H12*C*	2.86	2 − *x*, 1 − *y*, 1 − *z*
H2*A*⋯H11*C*	2.36	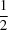 + *x*, 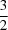 − *y*, 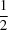 + *z*

**Table 4:** Percentage contributions of inter-atomic contacts to the Hirshfeld surfaces for (I)

Contact	percentage contribution
H⋯H	56.8
S⋯H/H⋯S	27.2
O⋯H/H⋯O	9.9
C⋯/H⋯C	4.0
N⋯H/H⋯N	1.1
Sn⋯S/S⋯Sn	0.5
S⋯S	0.5

**Table 5:** Summary of Sn—S, Sn⋯S distances (Å) in *R*_2_Sn(S_2_CN*RR*′)_2_ structures featuring secondary Sn⋯S inter­actions

*R*	*R*, *R*′	Sn—S_short_, Sn—S_long_	Sn⋯S	motif	Reference
Me	Et, Et	2.5174 (18), 2.961 (3); 2.528 (2), 2.9162 (17)	3.853 (2)	*A*	Morris & Schlemper (1979 ▸)
Me	(CH_2_CH_2_)Me	2.5367 (14), 2.9171 (16); 2.5577 (15), 2.8953 (16)	3.6978 (18)	*A*	Zia-ur-Rehman *et al.* (2007 ▸)
Me	(CH_2_CH_2_)O	2.5429 (6), 2.8923 (6); 2.5649 (7), 2.9137 (6)	3.5654 (7)	*A*	this work
C(H)=CH_2_	Cy	2.514 (5), 2.914 (4); 2.536 (4), 2.914 (4)	3.662 (5)	*A*	Hall & Tiekink (1998 ▸)
CH_2_Ph	Et, Et	2.5310 (11), 2.8940 (11); 2.5396 (10), 2.9109 (11)	3.8161 (12)	*A*	Yin *et al.* (2003 ▸)
CH_2_PhCl-2	(CH_2_CH_2_)NMe	2.5401 (13), 2.8050 (13); 2.5675 (13), 2.8675 (12)	3.9071 (13)	*A*	Yin & Xue (2005*a* ▸)
CH_2_PhCl-3*^*a*^*	(CH_2_CH_2_)NEt	2.520 (3), 2.840 (3); 2.556 (2), 2.893 (3)	3.638 (3)	*A*	Xue *et al.* (2005 ▸)
CH_2_PhCl-4	(CH_2_CH_2_)NMe	2.534 (2), 2.968 (3); 2.550 (2), 2.858 (3)	3.765 (3)	*A*	Yin & Xue (2005*b* ▸)
CH_2_PhCN-4	Et, Et	2.524 (3), 2.885 (3); 2.537 (2), 2.879 (2)	3.821 (3)	*A*	Yin & Xue (2006 ▸)
Me*^*b*^*	Et; CH_2_Ph	2.543 (2), 2.943 (2); 2.549 (2), 2.909 (2)	3.724 (3)	*B*	Barba *et al.* (2012 ▸)
		2.579 (2), 2.842 (2); 2.609 (2), 3.003 (2)	2.978 (5)*^*c*^*		
Me*^*d*^*	CH_2_Ph, 0.5(1,3-CH_2_C_6_H_4_CH_2_)	2.5086 (13), 2.8791 (15); 2.5217 (14), 3.1510 (16)	3.9641 (15)	*C*	Santacruz-Juárez *et al.* (2008 ▸)
Me^*d*,*e*^	bi­cyclo­[2.2.1]hept-2yl, 0.5(CH_2_)_4_	2.5179 (12), 2.9015 (13); 2.5321 (12), 2.9600 (13)	3.9453 (14)	*C*	Rojas-León *et al.* (2012 ▸)
Me*^*f*^*	(CH_2_)_2_^i^Pr, 0.5(1,3-CH_2_C_6_H_4_CH_2_)	2.5319 (18), 2.8855 (18); 2.5356 (17), 2.9663 (19)	4.0480 (19)	*C*′	Santacruz-Juárez *et al.* (2008 ▸)
		2.5306 (17), 2.9492 (19); 2.5402 (19), 2.9633 (19)	3.7050 (17)		

Notes: (*a*) piperazine mono-solvate; (*b*) two mol­ecules in the asymmetric unit; (*c*) Sn⋯N secondary inter­action; (*d*) the binuclear mol­ecule is located about a centre of inversion; (*e*) CDCl_3_ di-solvate per binuclear entity; (*f*) two mol­ecules in the asymmetric unit with each being located about a centre of inversion.

**Table 6:** Experimental details

Crystal data	
Chemical formula	[Sn(CH_3_)_2_(C_5_H_8_NOS_2_)_2_]
*M*_r_	473.24
Crystal system, space group	Monoclinic, *P*2_1_/*n*
Temperature (K)	100
*a*, *b*, *c* (Å)	10.1472 (1), 13.6653 (1), 13.8122 (1)
β (°)	104.959 (1)
*V* (Å^3^)	1850.36 (3)
*Z*	4
Radiation type	Cu *K*α
μ (mm^−1^)	15.25
Crystal size (mm)	0.24 × 0.09 × 0.06

Data collection	
Diffractometer	Agilent SuperNova, Dual, Cu at zero, AtlasS2
Absorption correction	Gaussian (*CrysAlis PRO*; Rigaku Oxford Diffraction, 2015 ▸)
*T*_min_, *T*_max_	0.242, 0.759
No. of measured, independent and observed [*I* > 2σ(*I*)] reflections	19588, 3865, 3809
*R*_int_	0.031
(sin θ/λ)_max_ (Å^−1^)	0.631

Refinement	
*R*[*F* ^2^ > 2σ(*F* ^2^)], *wR*(*F* ^2^), *S*	0.024, 0.065, 1.07
No. of reflections	3865
No. of parameters	192
H-atom treatment	H-atom parameters constrained
Δρ_max_, Δρ_min_ (e Å^−3^)	0.45, −0.50

Computer programs: *CrysAlis PRO* (Rigaku Oxford Diffraction, 2015 ▸), *SHELXS* (Sheldrick, 2008 ▸), *SHELXL2014* (Sheldrick, 2015 ▸), *ORTEP-3 for Windows* (Farrugia, 2012 ▸), *DIAMOND* (Brandenburg, 2006 ▸) and *publCIF* (Westrip, 2010 ▸).

## supplementary crystallographic information

### Crystal data

**Table:**

[Sn(CH_3_)_2_(C_5_H_8_NOS_2_)_2_]	*F*(000) = 952
*M**_r_* = 473.24	*D*_x_ = 1.699 Mg m^−^^3^
Monoclinic, *P*2_1_/*n*	Cu *K*α radiation, λ = 1.54184 Å
*a* = 10.1472 (1) Å	Cell parameters from 14936 reflections
*b* = 13.6653 (1) Å	θ = 3.2–76.6°
*c* = 13.8122 (1) Å	µ = 15.25 mm^−^^1^
β = 104.959 (1)°	*T* = 100 K
*V* = 1850.36 (3) Å^3^	Prism, colourless
*Z* = 4	0.24 × 0.09 × 0.06 mm

### Data collection

**Table:**

Agilent SuperNova, Dual, Cu at zero, AtlasS2 diffractometer	3865 independent reflections
Radiation source: micro-focus sealed X-ray tube, SuperNova (Cu) X-ray Source	3809 reflections with *I* > 2σ(*I*)
Mirror monochromator	*R*_int_ = 0.031
ω scans	θ_max_ = 76.8°, θ_min_ = 4.6°
Absorption correction: gaussian (CrysAlis PRO; Rigaku Oxford Diffraction, 2015)	*h* = −12→12
*T*_min_ = 0.242, *T*_max_ = 0.759	*k* = −13→17
19588 measured reflections	*l* = −17→17

### Refinement

**Table:**

Refinement on *F*^2^	0 restraints
Least-squares matrix: full	Hydrogen site location: inferred from neighbouring sites
*R*[*F*^2^ > 2σ(*F*^2^)] = 0.024	H-atom parameters constrained
*wR*(*F*^2^) = 0.065	*w* = 1/[σ^2^(*F*_o_^2^) + (0.0322*P*)^2^ + 2.5554*P*] where *P* = (*F*_o_^2^ + 2*F*_c_^2^)/3
*S* = 1.07	(Δ/σ)_max_ = 0.001
3865 reflections	Δρ_max_ = 0.45 e Å^−^^3^
192 parameters	Δρ_min_ = −0.50 e Å^−^^3^

### Special details

**Table:**

Geometry. All esds (except the esd in the dihedral angle between two l.s. planes) are estimated using the full covariance matrix. The cell esds are taken into account individually in the estimation of esds in distances, angles and torsion angles; correlations between esds in cell parameters are only used when they are defined by crystal symmetry. An approximate (isotropic) treatment of cell esds is used for estimating esds involving l.s. planes.

### Fractional atomic coordinates and isotropic or equivalent isotropic displacement parameters (Å^2^)

**Table:**

	*x*	*y*	*z*	*U*_iso_*/*U*_eq_	
Sn	0.54609 (2)	0.47196 (2)	0.20083 (2)	0.01811 (6)	
S1	0.60114 (7)	0.47340 (5)	0.39117 (5)	0.02266 (14)	
S2	0.76105 (7)	0.60660 (4)	0.29478 (4)	0.02164 (13)	
S3	0.36959 (7)	0.34005 (5)	0.20926 (4)	0.02368 (13)	
S4	0.38512 (7)	0.40224 (5)	0.00694 (4)	0.02491 (14)	
O1	0.9722 (2)	0.62761 (17)	0.68544 (15)	0.0315 (4)	
O2	−0.1073 (2)	0.28893 (17)	−0.02186 (16)	0.0334 (5)	
N1	0.7980 (2)	0.59004 (16)	0.49256 (16)	0.0216 (4)	
N2	0.1802 (2)	0.29886 (17)	0.04134 (16)	0.0235 (5)	
C1	0.7294 (3)	0.56096 (18)	0.40120 (18)	0.0188 (5)	
C2	0.8983 (3)	0.6702 (2)	0.5084 (2)	0.0245 (5)	
H2A	0.8543	0.7321	0.5205	0.029*	
H2B	0.9318	0.6786	0.4477	0.029*	
C3	1.0169 (3)	0.6474 (2)	0.5975 (2)	0.0294 (6)	
H3A	1.0670	0.5899	0.5817	0.035*	
H3B	1.0805	0.7036	0.6103	0.035*	
C4	0.8853 (3)	0.5444 (2)	0.6688 (2)	0.0285 (6)	
H4A	0.8579	0.5290	0.7309	0.034*	
H4B	0.9355	0.4874	0.6522	0.034*	
C5	0.7594 (3)	0.5622 (2)	0.58459 (19)	0.0253 (5)	
H5A	0.7031	0.5021	0.5722	0.030*	
H5B	0.7044	0.6151	0.6037	0.030*	
C6	0.2990 (3)	0.34278 (18)	0.07953 (18)	0.0202 (5)	
C7	0.1020 (3)	0.2459 (2)	0.1005 (2)	0.0267 (6)	
H7A	0.1494	0.2502	0.1726	0.032*	
H7B	0.0943	0.1760	0.0810	0.032*	
C8	−0.0379 (3)	0.2905 (2)	0.0819 (2)	0.0310 (6)	
H8A	−0.0919	0.2539	0.1201	0.037*	
H8B	−0.0296	0.3590	0.1061	0.037*	
C9	−0.0330 (3)	0.3430 (2)	−0.0777 (2)	0.0303 (6)	
H9A	−0.0261	0.4120	−0.0551	0.036*	
H9B	−0.0829	0.3418	−0.1495	0.036*	
C10	0.1087 (3)	0.3021 (2)	−0.06570 (19)	0.0249 (5)	
H10A	0.1028	0.2354	−0.0945	0.030*	
H10B	0.1599	0.3439	−0.1020	0.030*	
C11	0.4147 (3)	0.5961 (2)	0.1692 (2)	0.0260 (5)	
H11A	0.4573	0.6513	0.2110	0.039*	
H11B	0.3275	0.5803	0.1837	0.039*	
H11C	0.3987	0.6137	0.0983	0.039*	
C12	0.7041 (3)	0.38945 (19)	0.1667 (2)	0.0242 (5)	
H12A	0.7154	0.4095	0.1012	0.036*	
H12B	0.6809	0.3197	0.1650	0.036*	
H12C	0.7894	0.4008	0.2181	0.036*	

### Atomic displacement parameters (Å^2^)

**Table:**

	*U*^11^	*U*^22^	*U*^33^	*U*^12^	*U*^13^	*U*^23^
Sn	0.02190 (10)	0.01935 (10)	0.01260 (9)	0.00090 (6)	0.00357 (6)	−0.00052 (5)
S1	0.0275 (3)	0.0258 (3)	0.0140 (3)	−0.0074 (2)	0.0041 (2)	−0.0009 (2)
S2	0.0290 (3)	0.0213 (3)	0.0154 (3)	−0.0032 (2)	0.0069 (2)	−0.0006 (2)
S3	0.0290 (3)	0.0286 (3)	0.0117 (3)	−0.0060 (2)	0.0022 (2)	0.0025 (2)
S4	0.0282 (3)	0.0332 (3)	0.0131 (3)	−0.0068 (3)	0.0051 (2)	0.0014 (2)
O1	0.0294 (10)	0.0400 (11)	0.0208 (9)	−0.0022 (9)	−0.0013 (8)	−0.0006 (8)
O2	0.0246 (10)	0.0416 (12)	0.0313 (11)	−0.0046 (9)	0.0025 (8)	−0.0013 (9)
N1	0.0257 (11)	0.0236 (10)	0.0149 (10)	−0.0024 (9)	0.0041 (8)	−0.0016 (8)
N2	0.0258 (11)	0.0271 (11)	0.0163 (10)	−0.0043 (9)	0.0033 (9)	0.0012 (9)
C1	0.0234 (12)	0.0153 (11)	0.0168 (11)	0.0006 (9)	0.0036 (9)	−0.0018 (9)
C2	0.0249 (13)	0.0256 (13)	0.0201 (12)	−0.0042 (10)	0.0007 (10)	−0.0027 (10)
C3	0.0248 (13)	0.0337 (15)	0.0273 (14)	0.0014 (11)	0.0023 (11)	0.0002 (12)
C4	0.0323 (15)	0.0301 (14)	0.0208 (13)	0.0020 (12)	0.0029 (11)	0.0029 (11)
C5	0.0291 (14)	0.0320 (14)	0.0140 (11)	−0.0044 (11)	0.0040 (10)	−0.0019 (10)
C6	0.0233 (12)	0.0204 (11)	0.0160 (11)	0.0013 (9)	0.0037 (9)	−0.0002 (9)
C7	0.0293 (14)	0.0290 (13)	0.0212 (12)	−0.0091 (11)	0.0053 (11)	0.0022 (11)
C8	0.0284 (14)	0.0373 (15)	0.0278 (14)	−0.0074 (12)	0.0083 (11)	−0.0038 (12)
C9	0.0300 (14)	0.0314 (15)	0.0253 (13)	−0.0019 (12)	−0.0002 (11)	−0.0020 (11)
C10	0.0265 (13)	0.0295 (13)	0.0161 (12)	−0.0074 (11)	0.0010 (10)	−0.0027 (10)
C11	0.0279 (13)	0.0244 (13)	0.0260 (13)	0.0111 (11)	0.0074 (11)	0.0042 (10)
C12	0.0264 (13)	0.0222 (12)	0.0228 (12)	0.0052 (10)	0.0043 (10)	−0.0042 (10)

### Geometric parameters (Å, º)

**Table:**

Sn—S1	2.5429 (6)	C3—H3A	0.9900
Sn—S2	2.8923 (6)	C3—H3B	0.9900
Sn—S3	2.5649 (7)	C4—C5	1.509 (4)
Sn—S4	2.9137 (6)	C4—H4A	0.9900
Sn—C11	2.132 (3)	C4—H4B	0.9900
Sn—C12	2.111 (3)	C5—H5A	0.9900
C1—S1	1.747 (3)	C5—H5B	0.9900
C1—S2	1.702 (3)	C7—C8	1.505 (4)
C6—S3	1.750 (3)	C7—H7A	0.9900
C6—S4	1.697 (3)	C7—H7B	0.9900
O1—C4	1.421 (4)	C8—H8A	0.9900
O1—C3	1.428 (4)	C8—H8B	0.9900
O2—C9	1.418 (4)	C9—C10	1.512 (4)
O2—C8	1.424 (4)	C9—H9A	0.9900
N1—C1	1.335 (3)	C9—H9B	0.9900
N1—C2	1.472 (3)	C10—H10A	0.9900
N1—C5	1.473 (3)	C10—H10B	0.9900
N2—C6	1.328 (4)	C11—H11A	0.9800
N2—C7	1.468 (3)	C11—H11B	0.9800
N2—C10	1.470 (3)	C11—H11C	0.9800
C2—C3	1.515 (4)	C12—H12A	0.9800
C2—H2A	0.9900	C12—H12B	0.9800
C2—H2B	0.9900	C12—H12C	0.9800
			
S1—Sn—S2	65.935 (19)	H4A—C4—H4B	108.0
S1—Sn—S3	85.878 (19)	N1—C5—C4	110.3 (2)
S1—Sn—S4	150.95 (2)	N1—C5—H5A	109.6
S1—Sn—C11	99.49 (8)	C4—C5—H5A	109.6
S1—Sn—C12	104.96 (8)	N1—C5—H5B	109.6
S2—Sn—S3	151.798 (18)	C4—C5—H5B	109.6
S2—Sn—S4	143.066 (18)	H5A—C5—H5B	108.1
S2—Sn—C11	86.87 (8)	N2—C6—S4	122.32 (19)
S2—Sn—C12	84.93 (8)	N2—C6—S3	119.1 (2)
S3—Sn—S4	65.137 (18)	S4—C6—S3	118.56 (15)
S3—Sn—C12	102.37 (8)	N2—C7—C8	109.1 (2)
S3—Sn—C11	99.28 (8)	N2—C7—H7A	109.9
S4—Sn—C11	84.20 (8)	C8—C7—H7A	109.9
S4—Sn—C12	84.15 (7)	N2—C7—H7B	109.9
C11—Sn—C12	148.24 (11)	C8—C7—H7B	109.9
C1—S1—Sn	92.73 (8)	H7A—C7—H7B	108.3
C1—S2—Sn	82.29 (9)	O2—C8—C7	111.4 (2)
C6—S3—Sn	92.55 (9)	O2—C8—H8A	109.3
C6—S4—Sn	82.27 (9)	C7—C8—H8A	109.3
C4—O1—C3	109.5 (2)	O2—C8—H8B	109.3
C9—O2—C8	110.2 (2)	C7—C8—H8B	109.3
C1—N1—C2	122.2 (2)	H8A—C8—H8B	108.0
C1—N1—C5	123.3 (2)	O2—C9—C10	111.8 (2)
C2—N1—C5	113.0 (2)	O2—C9—H9A	109.2
C6—N2—C7	124.6 (2)	C10—C9—H9A	109.2
C6—N2—C10	123.2 (2)	O2—C9—H9B	109.2
C7—N2—C10	112.2 (2)	C10—C9—H9B	109.2
N1—C1—S2	122.6 (2)	H9A—C9—H9B	107.9
N1—C1—S1	118.35 (19)	N2—C10—C9	109.3 (2)
S2—C1—S1	119.04 (14)	N2—C10—H10A	109.8
N1—C2—C3	110.0 (2)	C9—C10—H10A	109.8
N1—C2—H2A	109.7	N2—C10—H10B	109.8
C3—C2—H2A	109.7	C9—C10—H10B	109.8
N1—C2—H2B	109.7	H10A—C10—H10B	108.3
C3—C2—H2B	109.7	Sn—C11—H11A	109.5
H2A—C2—H2B	108.2	Sn—C11—H11B	109.5
O1—C3—C2	111.7 (2)	H11A—C11—H11B	109.5
O1—C3—H3A	109.3	Sn—C11—H11C	109.5
C2—C3—H3A	109.3	H11A—C11—H11C	109.5
O1—C3—H3B	109.3	H11B—C11—H11C	109.5
C2—C3—H3B	109.3	Sn—C12—H12A	109.5
H3A—C3—H3B	107.9	Sn—C12—H12B	109.5
O1—C4—C5	111.3 (2)	H12A—C12—H12B	109.5
O1—C4—H4A	109.4	Sn—C12—H12C	109.5
C5—C4—H4A	109.4	H12A—C12—H12C	109.5
O1—C4—H4B	109.4	H12B—C12—H12C	109.5
C5—C4—H4B	109.4		
			
C2—N1—C1—S2	5.5 (4)	C7—N2—C6—S4	179.6 (2)
C5—N1—C1—S2	171.0 (2)	C10—N2—C6—S4	−3.5 (4)
C2—N1—C1—S1	−173.9 (2)	C7—N2—C6—S3	−0.7 (4)
C5—N1—C1—S1	−8.4 (3)	C10—N2—C6—S3	176.2 (2)
Sn—S2—C1—N1	179.7 (2)	Sn—S4—C6—N2	168.6 (2)
Sn—S2—C1—S1	−0.98 (13)	Sn—S4—C6—S3	−11.13 (13)
Sn—S1—C1—N1	−179.5 (2)	Sn—S3—C6—N2	−167.2 (2)
Sn—S1—C1—S2	1.11 (15)	Sn—S3—C6—S4	12.56 (15)
C1—N1—C2—C3	−143.1 (3)	C6—N2—C7—C8	122.6 (3)
C5—N1—C2—C3	50.1 (3)	C10—N2—C7—C8	−54.6 (3)
C4—O1—C3—C2	61.3 (3)	C9—O2—C8—C7	−60.3 (3)
N1—C2—C3—O1	−55.1 (3)	N2—C7—C8—O2	57.4 (3)
C3—O1—C4—C5	−61.7 (3)	C8—O2—C9—C10	59.5 (3)
C1—N1—C5—C4	142.5 (3)	C6—N2—C10—C9	−123.5 (3)
C2—N1—C5—C4	−50.8 (3)	C7—N2—C10—C9	53.7 (3)
O1—C4—C5—N1	56.3 (3)	O2—C9—C10—N2	−55.8 (3)

### Hydrogen-bond geometry (Å, º)

Cg1 is the centroid of the (C8–C13) ring.

**Table:**

*D*—H···*A*	*D*—H	H···*A*	*D*···*A*	*D*—H···*A*
C10—H10*A*···S1^i^	0.99	2.86	3.809 (3)	161
C12—H12*C*···O1^ii^	0.98	2.47	3.399 (4)	158

Symmetry codes: (i) *x*−3/2, −*y*−1/2, *z*−3/2; (ii) −*x*+2, −*y*+1, −*z*+1.

## References

[bb1] Alcock, N. W. (1972). *Adv. Inorg. Chem. Radiochem* **15**, 1–58.

[bb2] Barba, V., Arenaza, B., Guerrero, J. & Reyes, R. (2012). *Heteroat. Chem.* **23**, 422–428.

[bb3] Bondi, A. (1964). *J. Phys. Chem.* **68**, 441–451.

[bb4] Brandenburg, K. (2006). *DIAMOND*. Crystal Impact GbR, Bonn, Germany.

[bb5] Farrugia, L. J. (2012). *J. Appl. Cryst.* **45**, 849–854.

[bb6] Ferreira, I. P., de Lima, G. M., Paniago, E. B., Rocha, W. R., Takahashi, J. A., Pinheiro, C. B. & Ardisson, J. D. (2012). *Eur. J. Med. Chem.* **58**, 493–503. 10.1016/j.ejmech.2012.10.021 23159807

[bb7] Ferreira, I. P., de Lima, G. M., Paniago, E. B., Rocha, W. R., Takahashi, J. A., Pinheiro, C. B. & Ardisson, J. D. (2014). *Polyhedron*, **79**, 161–169.

[bb8] Groom, C. R., Bruno, I. J., Lightfoot, M. P. & Ward, S. C. (2016). *Acta Cryst.* B**72**, 171–179. 10.1107/S2052520616003954 PMC4822653 27048719

[bb9] Hall, V. J. & Tiekink, E. R. T. (1998). *Main Group Met. Chem.* **21**, 245–254.

[bb10] Kevin, P., Lewis, D. J., Raftery, J., Malik, M. A. & O’Brien, P. (2015). *J. Cryst. Growth*, **415**, 93–99.

[bb11] Kim, K., Ibers, J. A., Jung, O.-S. & Sohn, Y. S. (1987). *Acta Cryst.* C**43**, 2317–2319.

[bb12] Marín-Luna, M., Alkorta, I. & Elguero, J. (2016). *J. Phys. Chem. A*, **120**, 648–656. 10.1021/acs.jpca.5b11876 26756083

[bb13] McKinnon, J. J., Jayatilaka, D. & Spackman, M. A. (2007). *Chem. Commun*. pp. 3814–3816. 10.1039/b704980c 18217656

[bb14] Mohamad, R., Awang, N., Jotani, M. M. & Tiekink, E. R. T. (2016). *Acta Cryst.* E**72**, 1130–1137. 10.1107/S2056989016011385 PMC4971856 27536397

[bb15] Mohamad, R., Awang, N., Kamaludin, N. F., Jotani, M. M. & Tiekink, E. R. T. (2017). *Acta Cryst.* E**73**, 260–265. 10.1107/S2056989017001098 PMC5290578 28217355

[bb22] Morris, J. S. & Schlemper, E. O. (1979). *J. Cryst. Mol. Struct* **9**, 13–31.

[bb16] Muthalib, A. F. A., Baba, I., Khaledi, H., Ali, H. M. & Tiekink, E. R. T. (2014). *Z. Kristallogr* **229**, 39–46.

[bb25] Ng, S. W., Wei, C., Kumar Das, V. G., Jameson, G. B. & Butcher, R. J. (1989). *J. Organomet. Chem* **365**, 75–82.

[bb17] Rigaku Oxford Diffraction (2015). *CrysAlis PRO*. Agilent Technologies Inc., Santa Clara, CA, USA.

[bb18] Rojas-León, I., Guerrero-Alvarez, J. A., Hernández-Paredes, J. & Höpfl, H. (2012). *Chem. Commun.* **48**, 401–403. 10.1039/c1cc15957g 22080365

[bb19] Santacruz-Juárez, E., Cruz-Huerta, J., Hernández-Ahuactzi, I. F., Reyes-Martínez, R., Tlahuext, H., Morales-Rojas, H. & Höpfl, H. (2008). *Inorg. Chem.* **47**, 9804–9812. 10.1021/ic801464s 18826217

[bb20] Sheldrick, G. M. (2008). *Acta Cryst.* A**64**, 112–122. 10.1107/S0108767307043930 18156677

[bb21] Sheldrick, G. M. (2015). *Acta Cryst.* C**71**, 3–8.

[bb23] Tiekink, E. R. T. (2008). *Appl. Organomet. Chem.* **22**, 533–550.

[bb24] Tiekink, E. R. T. (2017). *Coord. Chem. Rev.* http://dx.doi.org/10.1016/j.ccr.2017.01.009.

[bb26] Westrip, S. P. (2010). *J. Appl. Cryst.* **43**, 920–925.

[bb27] Xue, S., Yin, H., Wang, Q. & Wang, D. (2005). *Heteroat. Chem.* **16**, 271–277.

[bb28] Yin, H. D., Wang, C.-H., Wang, Y. & Ma, C.-L. (2003). *Chin. J. Chem* **21**, 356–360.

[bb29] Yin, H. D. & Xue, S. C. (2005*a*). *Appl. Organomet. Chem.* **19**, 194.

[bb30] Yin, H. D. & Xue, S. C. (2005*b*). *Appl. Organomet. Chem.* **19**, 187.

[bb31] Yin, H. D. & Xue, S. C. (2006). *Appl. Organomet. Chem.* **20**, 283–289.

[bb32] Yu, Y., Yang, H., Wei, Z.-W. & Tang, L.-F. (2014). *Heteroat. Chem.* **25**, 274–281.

[bb33] Zia-ur-Rehman, Shahzadi, S., Ali, S. & Jin, G.-X. (2007). *Turk. J. Chem* **31**, 435–442.

